# 4-(2-Azaniumyl­eth­yl)piperazin-1-ium bis(perchlorate)

**DOI:** 10.1107/S1600536811033976

**Published:** 2011-08-27

**Authors:** Mohammad Reza Reisi, Muhammad Saleh Salga, Hamid Khaledi, Hapipah Mohd Ali

**Affiliations:** a Chemistry Department, Isfahan University, 81646-73441 Isfahan, Iran; b Department of Chemistry, University of Malaya, 50603 Kuala Lumpur, Malaysia

## Abstract

In the title compound, C_6_H_17_N_3_^2+^·2ClO_4_^−^, the piperazine ring adopts a chair conformation with the ethyl­ammonium fragment occupying an equatorial position. In the crystal, the dications and perchlorate anions are linked through N—H⋯O hydrogen bonding and weak C—H⋯O hydrogen bonding into a three-dimensional supra­molecular network.

## Related literature

For the structures of related salts of the 4-(2-ammonio­ethyl)piperazin-1-ium cation, see: Guerfel *et al.* (1999 ▶); Srinivasan *et al.* (2008 ▶, 2009 ▶).
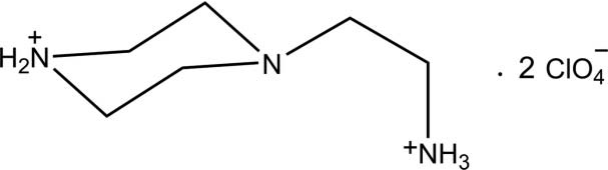


## Experimental

### 

#### Crystal data

C_6_H_17_N_3_^2+^·2ClO_4_^−^
*M* *_r_* = 330.13
Monoclinic, 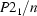

*a* = 7.5218 (1) Å
*b* = 11.4371 (2) Å
*c* = 15.2239 (2) Å
β = 97.437 (1)°
*V* = 1298.66 (3) Å^3^
*Z* = 4
Mo *K*α radiation
μ = 0.54 mm^−1^
*T* = 100 K
0.28 × 0.17 × 0.06 mm

#### Data collection

Bruker APEXII CCD diffractometer
Absorption correction: multi-scan (*SADABS*; Sheldrick, 1996 ▶) *T*_min_ = 0.863, *T*_max_ = 0.968
8644 measured reflections
2969 independent reflections
2671 reflections with *I* > 2σ(*I*)
*R*_int_ = 0.023

#### Refinement

*R*[*F* ^2^ > 2σ(*F* ^2^)] = 0.030
*wR*(*F* ^2^) = 0.080
*S* = 1.05
2969 reflections
187 parameters
5 restraints
H atoms treated by a mixture of independent and constrained refinement
Δρ_max_ = 0.27 e Å^−3^
Δρ_min_ = −0.59 e Å^−3^

### 

Data collection: *APEX2* (Bruker, 2007 ▶); cell refinement: *SAINT* (Bruker, 2007 ▶); data reduction: *SAINT*; program(s) used to solve structure: *SHELXS97* (Sheldrick, 2008 ▶); program(s) used to refine structure: *SHELXL97* (Sheldrick, 2008 ▶); molecular graphics: *X-SEED* (Barbour, 2001 ▶); software used to prepare material for publication: *SHELXL97* and *publCIF* (Westrip, 2010 ▶).

## Supplementary Material

Crystal structure: contains datablock(s) I, global. DOI: 10.1107/S1600536811033976/xu5296sup1.cif

Structure factors: contains datablock(s) I. DOI: 10.1107/S1600536811033976/xu5296Isup2.hkl

Supplementary material file. DOI: 10.1107/S1600536811033976/xu5296Isup3.cml

Additional supplementary materials:  crystallographic information; 3D view; checkCIF report

## Figures and Tables

**Table 1:** Hydrogen-bond geometry (Å, °)

*D*—H⋯*A*	*D*—H	H⋯*A*	*D*⋯*A*	*D*—H⋯*A*
N1—H1*C*⋯O4^i^	0.90 (2)	2.16 (2)	2.9298 (19)	143 (2)
N1—H1*D*⋯O3^ii^	0.88 (2)	2.09 (2)	2.964 (2)	168 (2)
N3—H3*C*⋯O6^i^	0.89 (2)	2.38 (2)	3.0741 (19)	135 (2)
N3—H3*C*⋯O4	0.89 (2)	2.39 (2)	3.0225 (19)	128 (2)
N3—H3*D*⋯O1^iii^	0.89 (2)	2.12 (2)	2.9875 (18)	163 (2)
N3—H3*E*⋯O8^iv^	0.88 (2)	2.14 (2)	2.9025 (19)	145 (2)
N3—H3*E*⋯O3	0.88 (2)	2.52 (2)	3.0724 (19)	122 (2)
C1—H1*B*⋯O7^v^	0.99	2.56	3.407 (2)	143
C3—H3*A*⋯O8^iii^	0.99	2.56	3.226 (2)	124
C5—H5*A*⋯O5^vi^	0.99	2.58	3.436 (2)	145
C5—H5*B*⋯O2^iii^	0.99	2.46	3.452 (2)	178

Symmetry codes: (i) 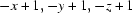; (ii) 
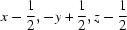
; (iii) 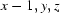; (iv) 
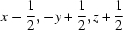
; (v) 
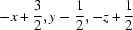
; (vi) 
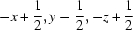
.

**Fig. 1.**
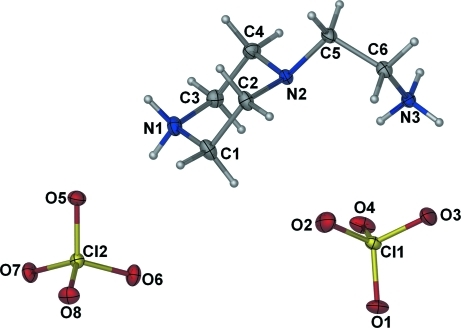
Molecular structure of the title compound with thermal ellipsoids at the 50% probability level. H atoms are drawn as spheres of arbitrary radius.

## supplementary crystallographic information

### Comment

The crystals of the title compound were obtained unexpectedly during an attempt to prepare a tin(IV) complex of 1-(2-aminoethyl)piperazine in the presence of sodium perchlorate. The organic molecule is doubly protonated at its primary and secondary N atoms, while the tertiary N atom, N2, remains unprotonated. Similar to the structures of some other 1-(2-ammoniumethyl)piperazinium salts (Guerfel *et al.*, 1999; Srinivasan *et al.*, 2008, 2009), the piperazine ring adopts a chair conformation with the ethylammonium group occupying an equatorial position. In the crystal, the dicationic organic moieties and perchlorate anions are linked through N—H···O and C—H···O interactions (Table 1) into a three-dimensional supra-molecular network.

### Experimental

A mixture of 4-(2-aminoethyl)piperazine (0.26 g, 2 mmol) and Bu_2_SnCl_2_ (0.6 g, 2 mmol) in methanol (50 ml) was refluxed for 2 h. NaClO_4_ (0.56 g, 4 mmol) was then added and the precipitated sodium chloride was filtered off. The filtrate was evaporated and the obtained solid was recrystallized from ethanol at room temperature to give the colorless crystals of the title compound.

### Refinement

The C-bound H atoms were placed at calculated positions and were treated as riding on their parent C atoms with C—H = 0.99 Å. The N-bound H atoms were located in a difference Fourier map, and refined with distance restraints of N—H = 0.91 (2) Å. For all H atoms, *U*_iso_(H) was set to 1.2*U*_eq_(carrier atom).

### Crystal data

**Table:**

C_6_H_17_N_3_^2+^·2ClO_4_^−^	*F*(000) = 688
*M**_r_* = 330.13	*D*_x_ = 1.688 Mg m^−^^3^
Monoclinic, *P*2_1_/*n*	Mo *K*α radiation, λ = 0.71073 Å
Hall symbol: -P 2yn	Cell parameters from 4052 reflections
*a* = 7.5218 (1) Å	θ = 2.2–30.5°
*b* = 11.4371 (2) Å	µ = 0.54 mm^−^^1^
*c* = 15.2239 (2) Å	*T* = 100 K
β = 97.437 (1)°	Blade, colourless
*V* = 1298.66 (3) Å^3^	0.28 × 0.17 × 0.06 mm
*Z* = 4	

### Data collection

**Table:**

Bruker APEXII CCD diffractometer	2969 independent reflections
Radiation source: fine-focus sealed tube	2671 reflections with *I* > 2σ(*I*)
graphite	*R*_int_ = 0.023
φ and ω scans	θ_max_ = 27.5°, θ_min_ = 2.2°
Absorption correction: multi-scan (*SADABS*; Sheldrick, 1996)	*h* = −9→9
*T*_min_ = 0.863, *T*_max_ = 0.968	*k* = −14→12
8644 measured reflections	*l* = −19→18

### Refinement

**Table:**

Refinement on *F*^2^	Primary atom site location: structure-invariant direct methods
Least-squares matrix: full	Secondary atom site location: difference Fourier map
*R*[*F*^2^ > 2σ(*F*^2^)] = 0.030	Hydrogen site location: inferred from neighbouring sites
*wR*(*F*^2^) = 0.080	H atoms treated by a mixture of independent and constrained refinement
*S* = 1.05	*w* = 1/[σ^2^(*F*_o_^2^) + (0.0388*P*)^2^ + 0.8608*P*] where *P* = (*F*_o_^2^ + 2*F*_c_^2^)/3
2969 reflections	(Δ/σ)_max_ = 0.001
187 parameters	Δρ_max_ = 0.27 e Å^−^^3^
5 restraints	Δρ_min_ = −0.59 e Å^−^^3^

### Special details

**Table:**

Geometry. All e.s.d.'s (except the e.s.d. in the dihedral angle between two l.s. planes) are estimated using the full covariance matrix. The cell e.s.d.'s are taken into account individually in the estimation of e.s.d.'s in distances, angles and torsion angles; correlations between e.s.d.'s in cell parameters are only used when they are defined by crystal symmetry. An approximate (isotropic) treatment of cell e.s.d.'s is used for estimating e.s.d.'s involving l.s. planes.
Refinement. Refinement of *F*^2^ against ALL reflections. The weighted *R*-factor *wR* and goodness of fit *S* are based on *F*^2^, conventional *R*-factors *R* are based on *F*, with *F* set to zero for negative *F*^2^. The threshold expression of *F*^2^ > σ(*F*^2^) is used only for calculating *R*-factors(gt) *etc*. and is not relevant to the choice of reflections for refinement. *R*-factors based on *F*^2^ are statistically about twice as large as those based on *F*, and *R*-factors based on ALL data will be even larger.

### Fractional atomic coordinates and isotropic or equivalent isotropic displacement parameters (Å^2^)

**Table:**

	*x*	*y*	*z*	*U*_iso_*/*U*_eq_	
N1	0.3280 (2)	0.44351 (13)	0.34193 (10)	0.0188 (3)	
H1C	0.390 (3)	0.5105 (15)	0.3389 (14)	0.023*	
H1D	0.268 (3)	0.4345 (19)	0.2884 (11)	0.023*	
N2	0.22434 (17)	0.25008 (12)	0.44167 (9)	0.0137 (3)	
N3	0.26916 (19)	0.16033 (12)	0.61553 (9)	0.0138 (3)	
H3C	0.308 (3)	0.2308 (14)	0.6010 (13)	0.017*	
H3D	0.161 (2)	0.1729 (17)	0.6323 (13)	0.017*	
H3E	0.340 (2)	0.1314 (17)	0.6606 (11)	0.017*	
C1	0.4538 (2)	0.34346 (15)	0.36621 (12)	0.0212 (4)	
H1A	0.5295	0.3604	0.4229	0.025*	
H1B	0.5334	0.3327	0.3199	0.025*	
C2	0.3466 (2)	0.23325 (15)	0.37506 (11)	0.0182 (3)	
H2A	0.2769	0.2137	0.3173	0.022*	
H2B	0.4289	0.1674	0.3928	0.022*	
C3	0.1959 (2)	0.45727 (15)	0.40696 (12)	0.0186 (3)	
H3A	0.1099	0.5205	0.3873	0.022*	
H3B	0.2595	0.4787	0.4658	0.022*	
C4	0.0963 (2)	0.34326 (15)	0.41350 (12)	0.0190 (3)	
H4A	0.0095	0.3513	0.4568	0.023*	
H4B	0.0289	0.3235	0.3552	0.023*	
C5	0.1375 (2)	0.13996 (15)	0.46127 (11)	0.0165 (3)	
H5A	0.1257	0.0886	0.4085	0.020*	
H5B	0.0160	0.1559	0.4768	0.020*	
C6	0.2498 (2)	0.08004 (14)	0.53790 (11)	0.0158 (3)	
H6A	0.1911	0.0065	0.5529	0.019*	
H6B	0.3694	0.0606	0.5214	0.019*	
Cl1	0.72577 (5)	0.21252 (3)	0.61138 (3)	0.01391 (10)	
O1	0.90644 (15)	0.24485 (11)	0.64617 (9)	0.0220 (3)	
O2	0.71413 (18)	0.18723 (12)	0.51861 (8)	0.0263 (3)	
O3	0.67246 (17)	0.10996 (11)	0.65736 (9)	0.0241 (3)	
O4	0.60538 (16)	0.30697 (11)	0.62494 (10)	0.0246 (3)	
Cl2	0.74537 (5)	0.57603 (3)	0.26377 (2)	0.01355 (10)	
O5	0.59033 (15)	0.51100 (11)	0.22387 (8)	0.0188 (3)	
O6	0.75093 (17)	0.57359 (11)	0.35894 (8)	0.0200 (3)	
O7	0.73342 (17)	0.69479 (10)	0.23263 (8)	0.0204 (3)	
O8	0.90557 (15)	0.52178 (11)	0.23899 (8)	0.0202 (3)	

### Atomic displacement parameters (Å^2^)

**Table:**

	*U*^11^	*U*^22^	*U*^33^	*U*^12^	*U*^13^	*U*^23^
N1	0.0269 (8)	0.0140 (7)	0.0159 (7)	−0.0024 (6)	0.0044 (6)	0.0008 (6)
N2	0.0141 (6)	0.0131 (7)	0.0144 (7)	−0.0002 (5)	0.0034 (5)	0.0015 (5)
N3	0.0150 (6)	0.0119 (7)	0.0147 (7)	−0.0003 (5)	0.0026 (5)	0.0003 (5)
C1	0.0235 (9)	0.0174 (9)	0.0246 (9)	0.0005 (7)	0.0107 (7)	0.0029 (7)
C2	0.0227 (8)	0.0158 (8)	0.0178 (8)	−0.0002 (6)	0.0090 (6)	0.0002 (6)
C3	0.0193 (8)	0.0171 (8)	0.0195 (8)	0.0022 (6)	0.0030 (6)	0.0004 (7)
C4	0.0151 (8)	0.0187 (9)	0.0227 (9)	0.0016 (6)	0.0009 (6)	0.0016 (7)
C5	0.0175 (8)	0.0162 (8)	0.0158 (8)	−0.0044 (6)	0.0027 (6)	−0.0009 (6)
C6	0.0190 (8)	0.0125 (8)	0.0164 (8)	−0.0014 (6)	0.0046 (6)	−0.0026 (6)
Cl1	0.01169 (18)	0.01297 (19)	0.0174 (2)	−0.00018 (13)	0.00309 (13)	−0.00125 (14)
O1	0.0125 (6)	0.0229 (7)	0.0300 (7)	−0.0028 (5)	0.0010 (5)	−0.0039 (5)
O2	0.0263 (7)	0.0354 (8)	0.0176 (7)	−0.0007 (6)	0.0045 (5)	−0.0055 (6)
O3	0.0219 (6)	0.0207 (7)	0.0290 (7)	−0.0045 (5)	0.0012 (5)	0.0090 (5)
O4	0.0155 (6)	0.0157 (6)	0.0437 (8)	0.0021 (5)	0.0076 (5)	−0.0067 (6)
Cl2	0.01502 (19)	0.01298 (19)	0.01291 (19)	0.00041 (13)	0.00284 (13)	−0.00077 (13)
O5	0.0160 (6)	0.0196 (6)	0.0206 (6)	−0.0037 (5)	0.0016 (5)	−0.0013 (5)
O6	0.0283 (7)	0.0194 (6)	0.0128 (6)	0.0033 (5)	0.0045 (5)	−0.0001 (5)
O7	0.0274 (7)	0.0134 (6)	0.0207 (6)	−0.0012 (5)	0.0038 (5)	0.0031 (5)
O8	0.0164 (6)	0.0227 (7)	0.0223 (7)	0.0022 (5)	0.0056 (5)	−0.0069 (5)

### Geometric parameters (Å, °)

**Table:**

N1—C3	1.499 (2)	C3—H3A	0.9900
N1—C1	1.500 (2)	C3—H3B	0.9900
N1—H1C	0.902 (15)	C4—H4A	0.9900
N1—H1D	0.884 (15)	C4—H4B	0.9900
N2—C4	1.463 (2)	C5—C6	1.513 (2)
N2—C5	1.467 (2)	C5—H5A	0.9900
N2—C2	1.467 (2)	C5—H5B	0.9900
N3—C6	1.489 (2)	C6—H6A	0.9900
N3—H3C	0.894 (15)	C6—H6B	0.9900
N3—H3D	0.894 (15)	Cl1—O2	1.4331 (13)
N3—H3E	0.878 (15)	Cl1—O1	1.4414 (12)
C1—C2	1.512 (2)	Cl1—O4	1.4416 (12)
C1—H1A	0.9900	Cl1—O3	1.4489 (13)
C1—H1B	0.9900	Cl2—O7	1.4376 (12)
C2—H2A	0.9900	Cl2—O6	1.4444 (12)
C2—H2B	0.9900	Cl2—O8	1.4481 (12)
C3—C4	1.513 (2)	Cl2—O5	1.4487 (12)
			
C3—N1—C1	111.60 (13)	H3A—C3—H3B	108.3
C3—N1—H1C	109.7 (13)	N2—C4—C3	109.53 (13)
C1—N1—H1C	110.3 (13)	N2—C4—H4A	109.8
C3—N1—H1D	108.6 (14)	C3—C4—H4A	109.8
C1—N1—H1D	111.6 (14)	N2—C4—H4B	109.8
H1C—N1—H1D	104.8 (19)	C3—C4—H4B	109.8
C4—N2—C5	113.04 (13)	H4A—C4—H4B	108.2
C4—N2—C2	109.92 (13)	N2—C5—C6	109.07 (13)
C5—N2—C2	111.30 (13)	N2—C5—H5A	109.9
C6—N3—H3C	111.2 (13)	C6—C5—H5A	109.9
C6—N3—H3D	109.1 (13)	N2—C5—H5B	109.9
H3C—N3—H3D	105.1 (18)	C6—C5—H5B	109.9
C6—N3—H3E	112.0 (13)	H5A—C5—H5B	108.3
H3C—N3—H3E	110.4 (18)	N3—C6—C5	108.67 (13)
H3D—N3—H3E	108.8 (18)	N3—C6—H6A	110.0
N1—C1—C2	109.35 (14)	C5—C6—H6A	110.0
N1—C1—H1A	109.8	N3—C6—H6B	110.0
C2—C1—H1A	109.8	C5—C6—H6B	110.0
N1—C1—H1B	109.8	H6A—C6—H6B	108.3
C2—C1—H1B	109.8	O2—Cl1—O1	110.39 (8)
H1A—C1—H1B	108.3	O2—Cl1—O4	109.44 (8)
N2—C2—C1	109.97 (14)	O1—Cl1—O4	109.57 (8)
N2—C2—H2A	109.7	O2—Cl1—O3	109.10 (8)
C1—C2—H2A	109.7	O1—Cl1—O3	109.69 (8)
N2—C2—H2B	109.7	O4—Cl1—O3	108.62 (8)
C1—C2—H2B	109.7	O7—Cl2—O6	109.97 (7)
H2A—C2—H2B	108.2	O7—Cl2—O8	109.73 (8)
N1—C3—C4	109.21 (14)	O6—Cl2—O8	109.63 (7)
N1—C3—H3A	109.8	O7—Cl2—O5	109.52 (7)
C4—C3—H3A	109.8	O6—Cl2—O5	109.14 (7)
N1—C3—H3B	109.8	O8—Cl2—O5	108.82 (7)
C4—C3—H3B	109.8		

### Hydrogen-bond geometry (Å, °)

**Table:**

*D*—H···*A*	*D*—H	H···*A*	*D*···*A*	*D*—H···*A*
N1—H1C···O4^i^	0.90 (2)	2.16 (2)	2.9298 (19)	143.(2)
N1—H1D···O3^ii^	0.88 (2)	2.09 (2)	2.964 (2)	168.(2)
N3—H3C···O6^i^	0.89 (2)	2.38 (2)	3.0741 (19)	135.(2)
N3—H3C···O4	0.89 (2)	2.39 (2)	3.0225 (19)	128.(2)
N3—H3D···O1^iii^	0.89 (2)	2.12 (2)	2.9875 (18)	163.(2)
N3—H3E···O8^iv^	0.88 (2)	2.14 (2)	2.9025 (19)	145.(2)
N3—H3E···O3	0.88 (2)	2.52 (2)	3.0724 (19)	122.(2)
C1—H1B···O7^v^	0.99	2.56	3.407 (2)	143.
C3—H3A···O8^iii^	0.99	2.56	3.226 (2)	124.
C5—H5A···O5^vi^	0.99	2.58	3.436 (2)	145.
C5—H5B···O2^iii^	0.99	2.46	3.452 (2)	178.

Symmetry codes: (i) −*x*+1, −*y*+1, −*z*+1; (ii) *x*−1/2, −*y*+1/2, *z*−1/2; (iii) *x*−1, *y*, *z*; (iv) *x*−1/2, −*y*+1/2, *z*+1/2; (v) −*x*+3/2, *y*−1/2, −*z*+1/2; (vi) −*x*+1/2, *y*−1/2, −*z*+1/2.
